# Case Report: Immune checkpoint inhibitor plus chemotherapy benefited an elderly patient with non-small cell lung cancer following EGFR-TKI resistance

**DOI:** 10.3389/fonc.2026.1825651

**Published:** 2026-05-08

**Authors:** Zhiyu Liu, Chenchen Wei, Chongguo Zhang, Zhaoxia Wang

**Affiliations:** Department of Oncology, The Second Affiliated Hospital of Nanjing Medical University, Nanjing, Jiangsu, China

**Keywords:** chemotherapy, drug resistance, EGFR-positive, immunotherapy, NSCLC, renal metastasis

## Abstract

Renal metastasis from non-small cell lung cancer (NSCLC) is uncommon. This report describes an elderly patient with *EGFR*-mutant NSCLC and high PD-L1 expression (TPS 70%). Following six months of targeted therapy, the renal lesion increased in size compared to baseline. Although the patient remained asymptomatic, imaging and biopsy confirmed renal metastasis. Consequently, the treatment was switched to immunochemotherapy, resulting in tumor shrinkage and a durable response with stability lasting over 34 months. This case suggests that for elderly patients with advanced *EGFR*-mutant NSCLC and acquired resistance to EGFR-TKIs, individualized treatment strategies—guided by the resistance mechanism, PD-L1 expression level, and the patient’s specific circumstances—may maximize therapeutic outcomes. However, the mechanism by which tumors harboring high PD-L1 expression and *EGFR* p.L858R mutations continue to regress and maintain stable disease after switching to chemoimmunotherapy is not fully understood. Therefore, defining the specific subgroups most likely to benefit and identifying robust predictive biomarkers remain crucial for future clinical practice.

## 1 Introduction

*EGFR* mutations represent the most common driver alterations in NSCLC. The National Comprehensive Cancer Network (NCCN) Clinical Practice Guidelines in Oncology recommend osimertinib as the preferred first-line treatment for patients with metastatic NSCLC and *EGFR*-sensitizing mutations (1). Osimertinib extends the median progression-free survival (PFS) to 19 months and effectively overcomes T790M-mediated acquired resistance to first- and second-generation EGFR-TKIs (2).

This article reports a case of an elderly patient with *EGFR*-mutant NSCLC and high PD-L1 expression (TPS 70%). Upon acquiring resistance to targeted therapy, the patient exhibited enlargement of the renal lesion, which was subsequently diagnosed as metastasis through imaging and biopsy. This case suggests that in the context of EGFR-TKI resistance and high PD-L1 expression, the combination of immunotherapy and chemotherapy may be a potential treatment option. However, this observation remains hypothetical. Further research is needed to define which patient subtypes may benefit from this approach and to validate the value of PD-L1 as a predictive biomarker. This article aims to provide insights into dose adjustment strategies for elderly patients with advanced NSCLC, characterize the population with *EGFR* mutations who may benefit from immunotherapy, and highlight potential predictive markers.

## 2 Case presentation

**Figure 1:**
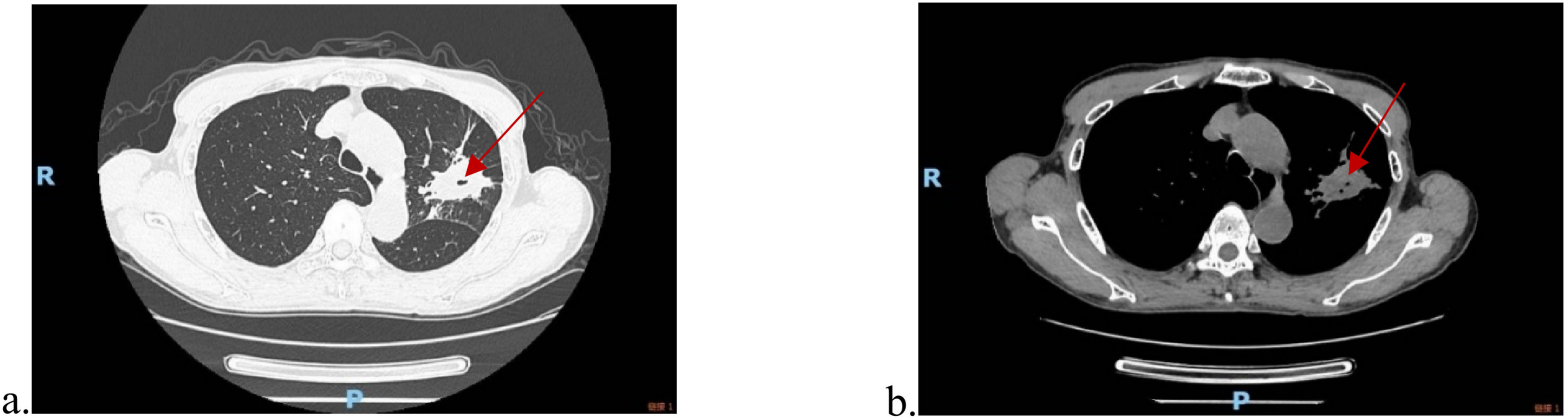
Patient CT at initial diagnosis **(a)** and contrast-enhanced CT **(b)**.

An 80-year-old male presented with cough and sputum production on March 24, 2022. Chest computed tomography (CT) revealed a mass in the left upper lobe (**Figure 1**), multiple bilateral pulmonary nodules, and hypodense lesions adjacent to the liver, right kidney, and right renal pelvis. Laboratory investigations demonstrated a positive PPD test and a positive T-SPOT.TB assay. The comprehensive tuberculosis antibody panel was positive for both tuberculosis IgG and LAM antibodies. Conversely, sputum smear microscopy revealed no acid-fast bacilli. Based on these findings, a diagnosis of pulmonary tuberculosis was established, and oral anti-tuberculosis therapy was initiated in April 2022.

On June 21, 2022, a chest CT scan at an outside hospital revealed a suspicious malignant mass in the left upper lobe. The following day, the patient underwent video-assisted thoracoscopic surgery (VATS) left upper lobe segmentectomy and lysis of thoracic adhesions. Histopathological examination of the resected specimen revealed an undifferentiated malignant carcinoma characterized by round, oval, and short spindle-shaped cells with associated necrosis. Immunohistochemical (IHC) staining was positive for TTF-1 (++), pan-cytokeratin (+++), and Vimentin (+++), with focal positivity for p63 and CK5/6. Conversely, markers for Calretinin, CD31, CD34, Desmin, Fli-1, HMB45, MelanA, Napsin-A, and SMA were negative. The Ki-67 proliferation index was approximately 80%. Next-generation sequencing (NGS) of the tumor tissue identified an *EGFR* L858R mutation in exon 21, alongside concomitant mutations in *FANCA* and TP53 (**Table 1**). The tumor exhibited a PD-L1 Tumor Proportion Score (TPS) of 70%, a Tumor Mutational Burden (TMB) of 6.18 mutations/Mb, and microsatellite stability (MSS). The final diagnosis was stage T2bNxMx left-sided non-small cell lung cancer (NSCLC) (according to the 8th edition of the TNM staging system).

**Table 1:** Mutation detected by NGS. Library preparation and sequencing were performed on FFPE samples using the MGISEQ-2000 platform on July 14, 2022.

Genes	Mutations site	Mutations type	Mutations frequency
EGFR	Exon21	p.L858R	86.02%
FANCA	Exon2	p.S236Ffs*2	--
TP53	Exon9	p.Q331*	24.27%

*indicates a nonsense mutation.

The patient presented to our institution on July 20, 2022. Contrast-enhanced CT of the chest and abdomen revealed postoperative changes consistent with prior left lung cancer resection and identified a new nodule in the posterior segment of the right upper lobe. Additionally, multiple delayed-enhancing lesions of indeterminate etiology were noted in both kidneys and perirenal areas; the largest lesion, located in the right kidney, measured approximately 2.0 cm × 2.4 cm. Subsequent non-contrast and contrast-enhanced MRI of the abdomen demonstrated abnormal signals in the renal parenchyma and pelvis, raising suspicion for either metastatic disease or infectious etiology. Although metastasis was considered a primary differential diagnosis, the patient declined a needle biopsy following consultation. The clinical diagnosis remained postoperative stage T2bNxMx NSCLC of the left lung, characterized by an *EGFR* exon 21 L858R mutation with concomitant *FANCA* and *TP53* mutations, and a PD-L1 Tumor Proportion Score (TPS) of 70%. Given that NGS of the primary lesion indicated an *EGFR* mutation, we initiated postoperative targeted therapy with osimertinib (80 mg, once daily). A follow-up contrast-enhanced CT performed on August 29, 2022—one month after treatment initiation—revealed stable postoperative changes and a reduction in the size of the right renal lesion to approximately 1.4 cm × 1.2 cm, alongside the persistent pulmonary nodule.

**Figure 2:**
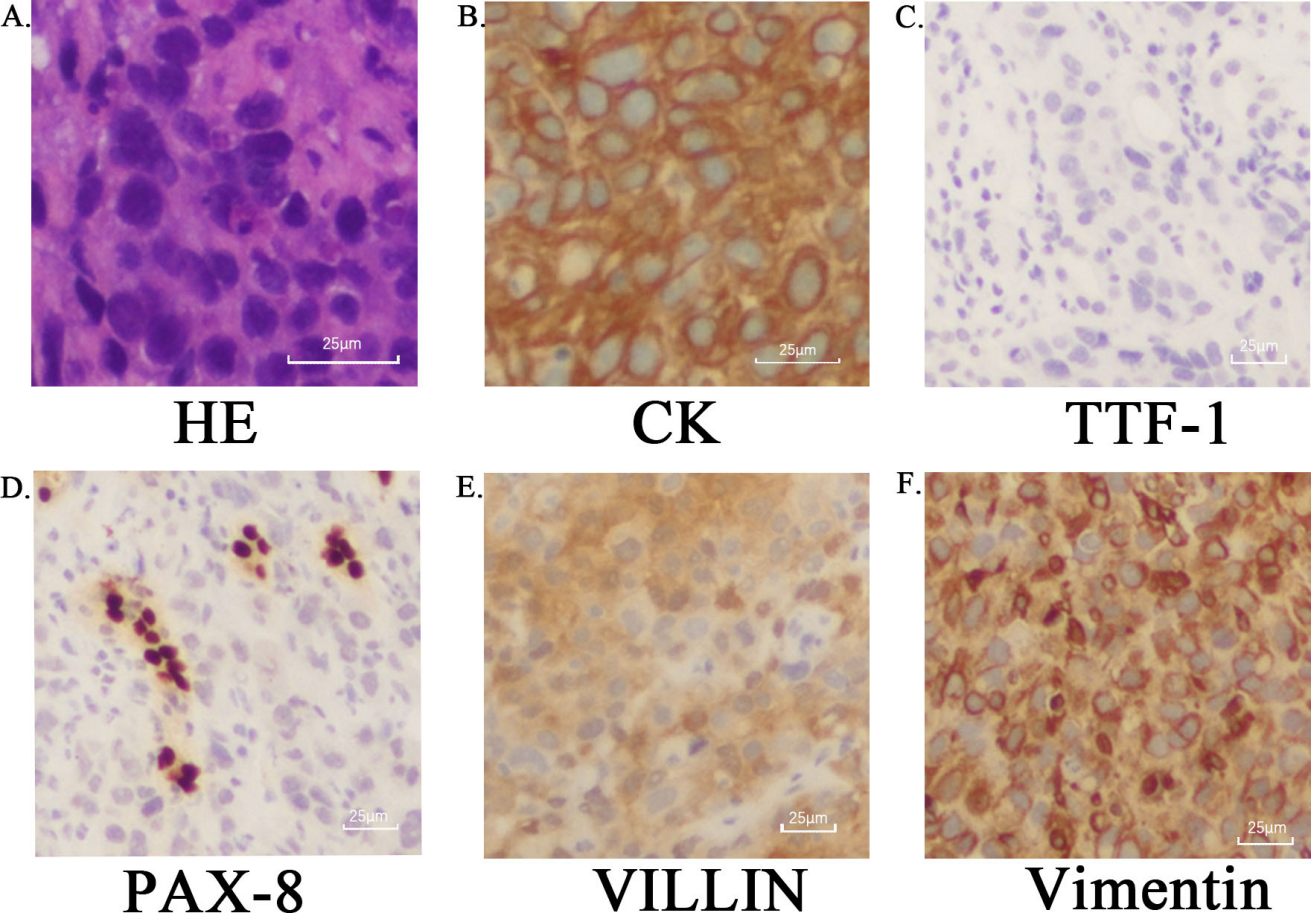
Histopathological and immunohistochemical features of renal metastasis from lung adenocarcinoma: The figure illustrates the microscopic appearance of a renal metastasis from lung adenocarcinoma. Hematoxylin and Eosin (H&E) staining reveals tumor cells arranged in nests and sheets with abundant cytoplasm and marked nuclear atypia **(A)**. Immunohistochemical staining demonstrates positivity for Cytokeratin (CK) **(B)** and negativity for TTF-1 **(C)**. Notably, the tumor cells are negative for PAX-8 **(D)**, while showing positivity for Villin **(E)** and Vimentin **(F)**.

Contrast-enhanced CT of the chest and abdomen performed on February 13, 2023, demonstrated multiple delayed-enhancing lesions involving the bilateral kidneys and renal pelves. The right renal lesion showed interval enlargement compared to prior imaging, measuring approximately 5.0 cm×4.8 cm, suggestive of metastasis. Following further communication with the patient, a renal biopsy was performed on March 1, 2023. Histopathological examination revealed a poorly differentiated carcinoma. Based on the clinical history and immunohistochemical profile, the diagnosis is consistent with poorly differentiated adenocarcinoma, with metastasis not excluded. Immunohistochemistry (**Figure 2**) demonstrated the following profile: CK7 (+), Villin (+), pan-CK (+), Vimentin (+), CK20 (focally +), p40 (scattered cells +), GATA-3 (focally +), CA9 (+/-), CD10 (focally +), RCC (–), Pax-8 (-), TTF-1 (-), Napsin A (-), PSA (-). The final diagnosis was Postoperative left lung adenocarcinoma (Stage T2bNxMx), with suspected renal metastasis. Further genetic testing was recommended; however, the patient declined due to financial constraints.

The patient’s surgical specimen demonstrated high PD-L1 expression, and secondary resistance developed following treatment with the EGFR-TKI osimertinib. The ATLANTIC study suggests that in heavily pretreated patients with advanced NSCLC, immunotherapy can elicit profound and durable clinical responses, particularly in the PD-L1 high-expression population. Furthermore, the ORIENT-31 study confirmed that a quadruplet regimen of “immunotherapy + antiangiogenic therapy + chemotherapy” significantly improved PFS. Based on the aforementioned evidence, such a combination was initially considered. However, taking into account the patient’s advanced age, history of drug resistance, tolerability concerns, and financial constraints—as well as the patient assistance program available for penpulimab—the regimen was ultimately modified to penpulimab (a PD-1 monoclonal antibody) combined with pemetrexed monotherapy.

Following two cycles of this regimen, a contrast-enhanced CT scan of the chest and abdomen on May 8, 2023, demonstrated significant tumor regression, with the lesion measuring approximately 3.2 cm × 2.8 cm. The overall response was evaluated as a partial response (PR) (**Table 2**), and maintenance therapy with the current regimen was initiated. To further evaluate the clinical status, PET-CT scans were performed on November 9, 2024, and December 6, 2025. The imaging demonstrated no evidence of local recurrence in the postoperative left lung, with no abnormal soft tissue shadows or increased FDG uptake observed. The right kidney exhibited irregular morphology with multiple hypermetabolic calculi; however, no focal abnormal density or metabolic activity suggestive of malignancy was detected. Additional findings included chronic inflammation in the right lower lobe and left lung, a calcified nodule in the right lower lobe, and scattered pulmonary micronodules (largest approximately 0.3 cm), which were considered likely chronic inflammatory in nature.

**Table 2:** Timeline of the relevant information.

Date	Examination/procedure	Lesion location & size (Long × Short axis)	Diagnosis	Efficacy evaluation	Treatment regimen
2022-03-04	PPD Test, TB Antibody Panel, Sputum AFB Smear, T-SPOT.TB	Left Upper Lobe (LUL) (4.6 cm × 2.4 cm)	Pulmonary Tuberculosis	—	Anti-tuberculosis Therapy
2022-06-21	Chest CT, Pathological Biopsy, NGS	LUL (4.6 cm × 2.4 cm)	Non-Small Cell Lung Cancer (NSCLC)	—	—
2022-07-25	Contrast-enhanced CT (Chest/Abdomen)	Bilateral Renal/Pelvic Lesions; Largest in Right Kidney (2.0 cm × 2.4 cm)	—	—	Osimertinib (80 mg, p.o., q.d.)
2022-08-29	Contrast-enhanced CT (Chest/Abdomen)	Bilateral Renal/Pelvic Lesions; Largest in Right Kidney (1.4 cm × 1.2 cm)	—	—	Osimertinib (80 mg, p.o., q.d.)
2023-02-13	Contrast-enhanced CT (Chest/Abdomen)	Bilateral Renal/Pelvic Lesions; Largest in Right Kidney (5.0 cm × 4.8 cm)	—	PD (Progressive Disease)	Osimertinib (80 mg, p.o., q.d.)
2023-03-01	Percutaneous Needle Biopsy	—	NSCLC with Renal Metastasis	—	Penpulimab (200 mg, d1) + Pemetrexed (0.7 g, d1)
2023-05-08	Contrast-enhanced CT (Chest/Abdomen)	Bilateral Renal/Pelvic Lesions; Largest in Right Kidney (3.2 cm × 2.8 cm)	—	PR (Partial Response)	Penpulimab (200 mg, d1) + Pemetrexed (0.7 g, d1)
2023-07-24 to 2024-01-03	Contrast-enhanced CT (Chest/Abdomen)	Bilateral Renal/Pelvic Lesions; Largest in Right Kidney (1.6 cm × 1.1 cm)	—	PR (Partial Response)	Penpulimab (200 mg, d1) + Pemetrexed (0.7 g, d1)
2024-01-25 to 2024-05-27	Contrast-enhanced CT (Chest/Abdomen)	Bilateral Renal/Pelvic Lesions; Largest in Right Kidney (1.4 cm × 1.0 cm)	—	SD (Stable Disease)	Penpulimab (200 mg, d1) + Pemetrexed (0.8 g, d1)
2024-11-08 to 2025-11-11	PET-CT	Lung/Kidneys: No abnormal soft tissue or FDG hypermetabolism detected.	—	SD (Stable Disease)	Penpulimab (200 mg, d1) + Pemetrexed (0.5 g, d1)
2025-12-06 to Present	PET-CT	Lung/Kidneys: No abnormal soft tissue or FDG hypermetabolism detected.	—	SD (Stable Disease)	Penpulimab (200 mg, d1) + Pemetrexed (0.5 g, d1)

**Figure 3:**
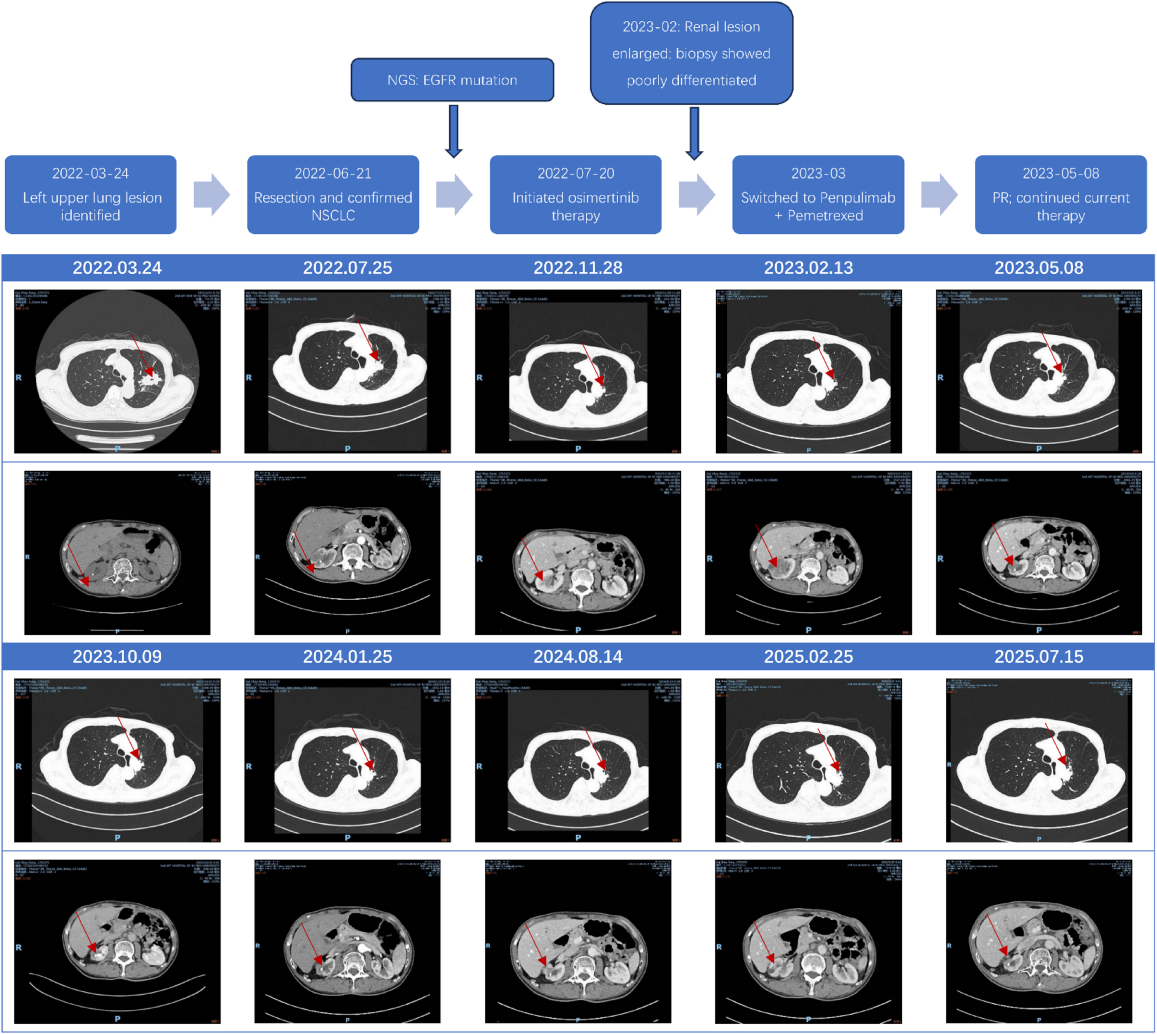
Timeline of diagnosis, treatment, and imaging follow-up: The upper panel illustrates a flowchart of key diagnostic and therapeutic milestones from March 2022 to May 2023. The lower panel displays axial CT images of the chest (top row) and abdomen (bottom row) obtained during the same period, with red arrows indicating the lesions.

The patient has maintained the current treatment with a PFS exceeding 34 months (**Figure 3**). Retrospective NGS analysis performed on May 2, 2025, using the patient’s kidney biopsy specimen from 2023, revealed *EGFR* and *MET* amplification (**Table 3**). While the MARIPOSA-2 study demonstrated that amivantamab combined with chemotherapy significantly improved PFS and objective response rate (ORR) in patients with EGFR-mutant advanced NSCLC following osimertinib resistance—prompting guidelines to include amivantamab as a standard second-line recommendation—the patient was not switched to this regimen. Theoretically, the presence of EGFR and MET amplification makes the patient a candidate for amivantamab therapy. However, given the drug’s safety profile and the patient’s favorable response to the current immunochemotherapy (with stable disease and absence of severe toxicities such as grade ≥3 immune-mediated pneumonitis or grade 4 myelosuppression), the current regimen was maintained. Switching to amivantamab-based combination therapy will be considered should disease progression occur.

**Table 3:** Mutation detected by NGS.

Genes	Mutations site	Mutations type	Mutations frequency
EGFR	Exon21	p.L858R	80.3%
MET	–	Amplification	11-fold increase
EGFR	–	Amplification	4.3-fold increase
TP53	Exon9	p.Q331*	25.4%

Library preparation and sequencing were performed on FFPE samples using the MGISEQ-2000 platform on May 2, 2025.

The asterisk (*) indicates a truncation mutation.

## 3 Discussion

EGFR mutations represent the most prevalent driver alteration in NSCLC. According to the NCCN Clinical Practice Guidelines in Oncology, osimertinib is recommended as the preferred first-line therapeutic option for patients with metastatic NSCLC harboring sensitizing *EGFR* mutations (1). This third-generation tyrosine kinase inhibitor (TKI) not only extends the median PFS to approximately 19 months but also demonstrates efficacy against the acquired *EGFR* T790M resistance mutation associated with prior first- and second-generation EGFR-TKI therapies (2). Consequently, we elected to initiate treatment with osimertinib as the primary management strategy for this patient. Unfortunately, despite this intervention, the patient exhibited enlargement of the renal lesion, which was subsequently confirmed as metastasis through imaging and biopsy 6 months following the initiation of therapy. We speculate that this clinical progression may represent acquired resistance to osimertinib. A retrospective study conducted in Japan suggests that in patients with *EGFR*-mutated NSCLC, a PD-L1 TPS of ≥20% may be associated with early resistance to osimertinib therapy (3). In this case, immunohistochemistry revealed a PD-L1 TPS of 70% in this patient, which may partially account for the early onset of resistance. While renal parenchymal metastasis is historically considered a rare event in NSCLC—with one large-scale study of 2,872 patients with stage IV disease reporting an incidence of approximately 0.87% (4)—the occurrence of such metastasis in this patient may be linked to the high invasiveness suggested by the immunohistochemical profile of the primary tumor. Furthermore, among patients harboring the *EGFR* L858R mutation, high PD-L1 expression has been associated with a significantly shorter mPFS compared to low or negative PD-L1 expression (5). This biological characteristic may also be a contributing factor to the early metastatic relapse observed in this patient.

Upon the development of osimertinib resistance, the patient declined repeat genetic testing. Given the high PD-L1 expression observed in the surgical specimen, combined with the patient’s advanced age and evidence from the ATLANTIC and ORIENT-31 trials, we initiated a personalized regimen comprising an immune checkpoint inhibitor(ICI) plus chemotherapy. The ATLANTIC study indicated that in patients with advanced NSCLC who had progressed on EGFR-TKIs, immunotherapy could still achieve significant and durable responses in those with high PD-L1 expression, demonstrating favorable clinical efficacy (6). Moreover, the ILLUMINATE study (7) demonstrated that among patients with EGFR T790M-negative disease who progressed after first-line osimertinib and received dual ICI plus chemotherapy, those with PD-L1 expression ≥50% achieved a significantly prolonged mPFS of 13.1 months. Based on the ORIENT-31 trial, this combination regimen has established a new standard of care for immunotherapy in NSCLC following EGFR-TKI failure. This is supported by the largest meta-analysis to date, which further confirmed the benefits of this four-drug combination, suggesting that early initiation of an “immunotherapy + anti-angiogenesis + chemotherapy” regimen can provide substantial clinical benefit for patients with extensive metastasis and no identified resistance mechanism after EGFR-TKI treatment. However, based on results from several clinical studies investigating the combination of EGFR-targeted agents and ICIs (8, 9), we observed that although this regimen demonstrates high anti-tumor activity in certain patients, it is associated with significant treatment-related toxicities and a markedly increased incidence of interstitial lung disease (ILD).

Given the patient’s advanced age (80 years), never-smoker status, and good performance status (ECOG 0–1)—along with the absence of CNS metastases confirmed by baseline brain MRI and significant comorbidities other than benign prostatic hyperplasia—we selected an immunochemotherapy regimen after targeted therapy failure, as he was deemed unsuitable for multi-agent chemotherapy. Elderly patients often exhibit reduced drug tolerance due to age-related decline in organ function and a high burden of comorbidities. Consequently, moderate dose reduction may confer greater clinical benefit in older adults with advanced lung cancer (10–12); however, a comprehensive geriatric assessment remains essential to guide individualized treatment selection. In this case, chemotherapy dosage was titrated based on performance status and tolerance. The treatment response was favorable: after two cycles of chemoimmunotherapy, the renal metastasis demonstrated significant shrinkage, achieving a Partial Response (PR). Following 16 months of continuous treatment, follow-up PET-CT revealed no residual disease or FDG-avid lesions in the left lung or right kidney.

These findings suggest that for elderly patients, timely dose adjustments tailored to resistance mechanisms, PD-L1 expression, and individual clinical profiles may yield superior outcomes. Furthermore, whether chemoimmunotherapy confers significant survival benefits for elderly patients with metastatic EGFR-mutant NSCLC who have developed resistance to EGFR-TKIs warrants further investigation. Prior studies, such as CheckMate-722 and KEYNOTE-789, have generally failed to demonstrate significant survival benefits with chemoimmunotherapy in this population. However, the CheckMate-722 trial indicated a trend toward improved PFS with chemoimmunotherapy versus chemotherapy alone in the subgroup of patients with tumor PD-L1 expression ≥50%, albeit with a limited sample size.

Emerging evidence suggests that in some EGFR-mutant tumors, acquired resistance to targeted therapy may coincide with a phenotypic transition of the tumor microenvironment from “cold” to “hot,” potentially sensitizing the tumor to subsequent immunotherapy (13, 14). In this case, the patient harbored an EGFR mutation and high PD-L1 expression (TPS 70%). Following osimertinib resistance, treatment with chemoimmunotherapy resulted in a marked reduction in tumor burden. Notably, follow-up imaging confirmed the absence of active disease in the primary and metastatic sites, consistent with sustained disease control and no evidence of other metastatic lesions. We hypothesize that this favorable outcome was driven by alterations in the tumor microenvironment in conjunction with high PD-L1 expression. This observation provides preliminary evidence that PD-L1 expression status may serve as a predictive biomarker for immunotherapy efficacy in elderly patients with EGFR-mutant NSCLC. However, this remains hypothetical, and its definitive value awaits confirmation in larger-scale studies.

## Data Availability

The datasets presented in this study can be found in online repositories. The names of the repository/repositories and accession number(s) can be found in the article/Supplementary Material.

## References

[1] Ettinger DS Wood DE Aisner DL Akerley W Bauman JR Bharat A . NCCN Guidelines® Insights: Non–small cell lung cancer, version 2.2023. J Natl Compr Cancer Network. (2023) 21:340–50. doi: 10.6004/jnccn.2023.0020. PMID: 37015337

[2] Ramalingam SS Yang J-H Lee CK Kurata T Kim D-W John T . Osimertinib as first-line treatment of EGFR mutation–positive advanced non–small-cell lung cancer. J Clin Oncol. (2018) 36:9. doi: 10.1200/JCO.2017.74.7576. PMID: 28841389

[3] Hamakawa Y Agemi Y Shiba A Ikeda T Higashi Y Aga M . Association of PD-L1 tumor proportion score ≥20% with early resistance to osimertinib in patients with EGFR-mutated NSCLC. Cancer Med. (2023) 12:17788–97. doi: 10.1002/cam4.6405. PMID: 37548381 PMC10523952

[4] Niu FY Zhou Q Yang JJ Zhong WZ Chen ZH Deng W . Distribution and prognosis of uncommon metastases from non-small cell lung cancer. BMC Cancer. (2016) 16:149. doi: 10.1186/s12885-016-2169-5. PMID: 26911831 PMC4766662

[5] Su S Dong ZY Xie Z Yan LX Li YF Su J . Strong programmed death ligand 1 expression predicts poor response and De Novo resistance to EGFR tyrosine kinase inhibitors among NSCLC patients with EGFR mutation. J Thorac Oncol Off Publ Int Assoc For Study Lung Cancer. (2018) 13:1668–75. doi: 10.1016/j.jtho.2018.07.016. PMID: 30056164

[6] Garassino MC Cho BC Kim JH Mazieres J Vansteenkiste J Lena H . Durvalumab as third-line or later treatment for advanced non-small-cell lung cancer (ATLANTIC): an open-label, single-arm, phase 2 study. Lancet Oncol. (2018) 19:521–36. doi: 10.1016/S1470-2045(18)30144-X. PMID: 29545095 PMC7771363

[7] Lee C Liao BC Subramaniam S Chiu CH Mersiades A Ho CC . OA09.04 ILLUMINATE: Efficacy and safety of durvalumab-tremelimumab and chemotherapy in EGFR mutant NSCLC following progression on EGFR inhibitors. J Thorac Oncol. (2023) 18. doi: 10.1016/j.jtho.2023.09.057. PMID: 38826717

[8] Ahn MJ Cho BC Ou X Walding A Dymond AW Ren S . Osimertinib plus durvalumab in patients with EGFR-mutated, advanced NSCLC: a phase 1b, open-label, multicenter trial. J Thorac Oncol Off Publ Int Assoc For Study Lung Cancer. (2022) 17:718–23. doi: 10.1016/j.jtho.2022.01.012. PMID: 35181499

[9] Yang JC Gadgeel SM Sequist LV Wu CL Papadimitrakopoulou VA Su WC . Pembrolizumab in combination with erlotinib or gefitinib as first-line therapy for advanced NSCLC with sensitizing EGFR mutation. J Thorac Oncol Off Publ Int Assoc For Study Lung Cancer. (2019) 14:553–9. doi: 10.1016/j.jtho.2018.11.028. PMID: 30529597

[10] Chinese Society of Clinical Oncology (CSCO) Elderly Oncology Committee . Expert consensus on the treatment of advanced lung cancer in elderly patients (2025 edition). Zhonghua zhong liu za zhi [Chinese J Oncology]. (2025) 47:575–98. doi: 10.3760/cma.j.cn112152-20250326-00128. PMID: 40545563

[11] Losanno T Gridelli C . First-line treatment of metastatic non-small cell lung cancer in the elderly. Curr Oncol Rep. (2021) 23:119. doi: 10.1007/s11912-021-01105-y. PMID: 34342732

[12] Nacci A Calvani N Rizzo P Fedele P Orlando L Schiavone P . When less is better: the safety and efficacy of reduced intensity gemcitabine in a difficult patient population with advanced non-small-cell lung cancer. Med Oncol (Northwood London England). (2013) 30:370. doi: 10.1007/s12032-012-0370-1. PMID: 23322519

[13] Chen Q Xia L Wang J Zhu S Wang J Li X . EGFR-mutant NSCLC may remodel TME from non-inflamed to inflamed through acquiring resistance to EGFR-TKI treatment. Lung Cancer (Amsterdam Netherlands). (2024) 192:107815. doi: 10.1016/j.lungcan.2024.107815. PMID: 38754276

[14] Kim S Koh J Kim TM Oh S Kim S Youk J . Remodeling of tumor microenvironments by EGFR tyrosine kinase inhibitors in EGFR-mutant non-small cell lung cancer. iScience. (2025) 28:111736. doi: 10.1016/j.isci.2024.111736. PMID: 39898038 PMC11787596

