# Supplementary material for: Case Report: Immune checkpoint inhibitor plus chemotherapy benefited an elderly patient with non-small cell lung cancer following EGFR-TKI resistance
Source: Front Oncol. 2026 May 8;16:1825651. doi: 10.3389/fonc.2026.1825651 (PMC13194002; doi:10.3389/fonc.2026.1825651)
Supplement: Supplementary file 1 [file DataSheet1.docx]

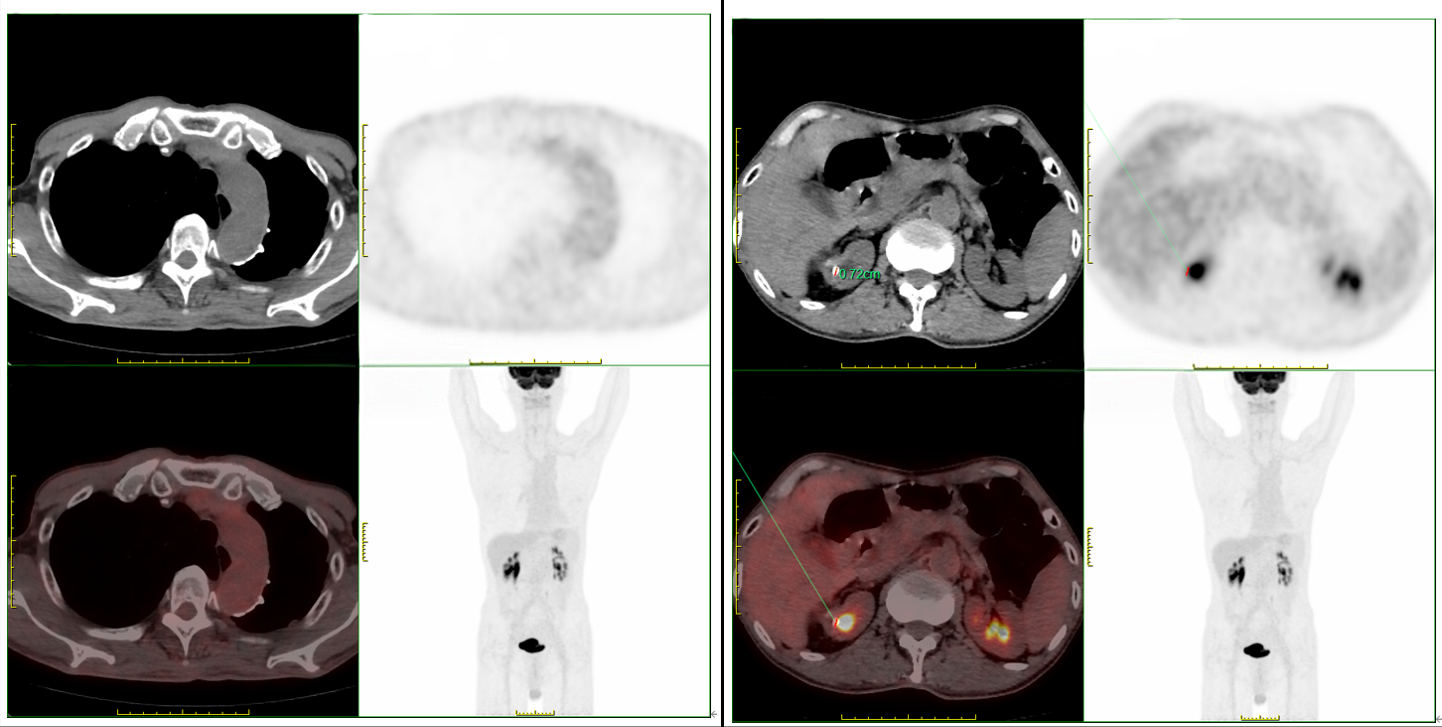


Supplementary Figure 1 Patient's PET-CT on November 9, 2024

**
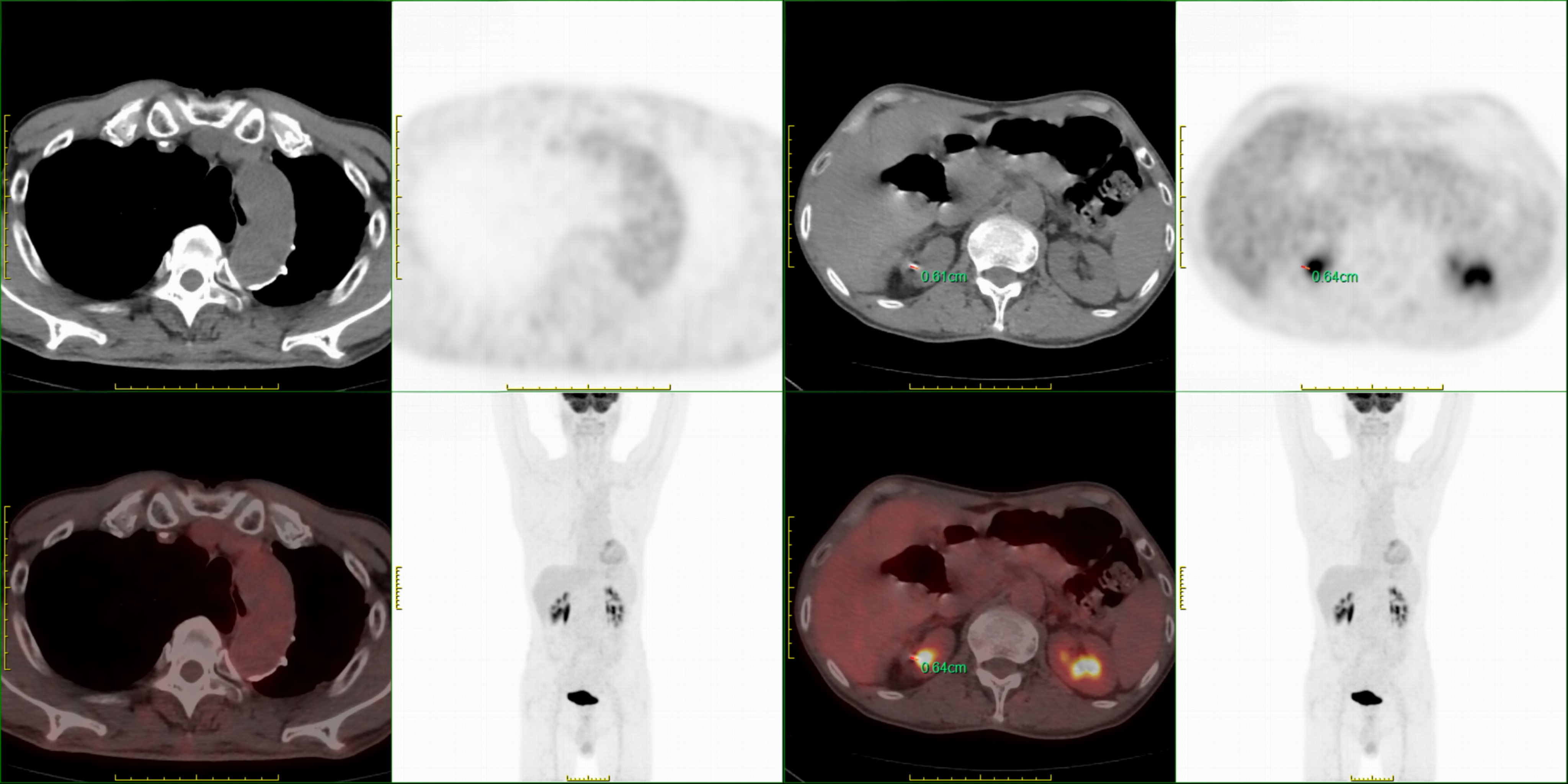
**

Supplementary Figures 2 Patient's PET-CT on December 6, 2025
